# Supplementary material for: The Long Non-Coding RNA H19 Drives the Proliferation of Diffuse Intrinsic Pontine Glioma with H3K27 Mutation
Source: Int J Mol Sci. 2021 Aug 25;22(17):9165. doi: 10.3390/ijms22179165 (PMC8431314; doi:10.3390/ijms22179165)
Supplement: Supplementary file 1 [file ijms-22-09165-s001.zip › Supplementary Table S1.pdf]

Supplementary Table S1. Clinical characteristics of DIPG cell models.

Cell line	Sex	Age	Sequence variation
**SU-DIPG-IV**	Female	2Y	HIST1H3B p.Lys28Met (c.83A>T) (H3.1K27M)
**VUMC-DIPG-A**	Female	3Y	H3F3A p.Lys28Met (c.83A>T) (H3.3K27M)
